# Invasive pseudomembranous upper airway and tracheal Aspergillosis refractory to systemic antifungal therapy and serial surgical debridement in an Immunocompetent patient

**DOI:** 10.1186/s12879-019-4744-2

**Published:** 2020-01-06

**Authors:** Shihan N. Khan, Rashmi Manur, John S. Brooks, Michael A. Husson, Kevin Leahy, Matthew Grant

**Affiliations:** 1 Department of Internal Medicine, Yale Infectious Diseases, Yale School of Medicine, PO Box 208022, New Haven, CT 06520 USA; 2 Department of Pathology and Laboratory Medicine, Perelman School of Medicine, Philadelphia, PA 19107 USA; 3 Department of Otorhinolaryngology: Head and Neck Surgery, Perelman School of Medicine, Philadelphia, PA 19107 USA

**Keywords:** Invasive aspergillosis, Upper airway, Antifungal therapy, Endoscopic debridement, Refractory disease

## Abstract

**Background:**

The development of respiratory infections secondary to *Aspergillus spp.* spores found ubiquitously in the ambient environment is uncommon in immunocompetent patients. Previous reports of invasive upper airway aspergillosis in immunocompetent patients have generally demonstrated the efficacy of treatment regimens utilizing antifungal agents in combination with periodic endoscopic debridement, with symptoms typically resolving within months of initiating therapy.

**Case presentation:**

A 43-year-old previously healthy female presented with worsening respiratory symptoms after failing to respond to long-term antibiotic treatment of bacterial sinusitis. Biopsy of her nasopharynx and trachea revealed extensive fungal infiltration and *Aspergillus fumigatus* was isolated on tissue culture. Several months of oral voriconazole monotherapy failed to resolve her symptoms and she underwent mechanical debridement for symptom control. Following transient improvement, her symptoms subsequently returned and failed to fully resolve in spite of increased voriconazole dosing and multiple additional tissue debridements over the course of many years.

**Conclusions:**

Invasive upper airway aspergillosis is exceedingly uncommon in immunocompetent patients. In the rare instances that such infections do occur, combinatorial voriconazole and endoscopic debridement is typically an efficacious treatment approach. However, some patients may continue to experience refractory symptoms. In such cases, continued aggressive treatment may potentially slow disease progression even if complete disease resolution cannot be achieved.

## Background

The development of chronic invasive aspergillosis of the upper airways (i.e. nasal passages, paranasal sinuses, pharynx) has historically been associated with patients suffering from some degree of underlying immunodeficiency, whether due to human immunodeficiency virus (HIV) infection, hematologic malignancy, or long-term treatment with immunosuppressive agents, amongst others [1]. Invasive aspergillosis is histologically characterized by invasion of airway mucosa by fungal hyphae that can additionally result in tissue ulceration or the formation of pseudomembranes consisting of necrotic epithelium overlying injured mucosa [2, 3]. Interestingly, while still exceedingly rare in otherwise healthy individuals, a growing body of literature in recent years indicates that a subset of the general population remains susceptible to *aspergillus*-related airway disease despite lacking the overt underlying pathologies or risk factors classically described in immunocompromised patients [4–7].

Immunocompetent patients suffering from invasive upper airway aspergillosis can be particularly challenging to diagnose given that many initially present with indolent sinus symptoms easily mistaken for bacterial sinusitis [6, 8]. Without initiation of treatment, these patients, not unlike their immunocompromised counterparts, can progress to more serious respiratory compromise resulting in poor outcomes. However, the majority will clinically improve with rapid symptom resolution and rare disease recurrence when treated appropriately with systemic antifungal agents in combination with surgical extirpation of diseased tissue [6]. This report describes a unique case of invasive pseudomembranous upper airway and tracheal aspergillosis presenting in an otherwise healthy female whose upper airway and respiratory symptoms proved to be refractory to aggressive antifungal treatment and numerous endoscopic debridements spanning the course of many years.

## Case presentation

A 43-year-old female with no significant past medical history presented with a chief complaint of worsening respiratory symptoms that included purulent nasal secretions, dysphagia, mild dysphonia and dyspnea with chronic cough. The symptoms had begun approximately 2 years previously and had failed to completely resolve in spite of multiple rounds of empiric antibiotic therapy for a presumed bacterial upper respiratory infection. She denied previous tobacco use and had no pertinent family history. Her physical exam was notable for erythematous nasal mucosa and a small anterior septal perforation. Blood work demonstrated a white blood cell count (WBC) of 11,400/uL (neutrophils 93.8%) and an erythrocyte sedimentation rate (ESR) of 11 mm/h. Electrolytes were all within normal limits and a random glucose was 96 mg/dL. Head and chest computed tomography (CT) showed air-fluid levels in the bilateral maxillary sinuses and irregular thickening along the left lateral and posterior tracheal wall just above the level of the medial clavicles but was negative for lung parenchymal changes.

Given these imaging findings in the setting of worsening dyspnea, the patient underwent a laryngoscopic examination, which revealed subglottic crusting with diffuse purulent secretions, anterolateral cricoid inflammation, and vocal cord inflammation. Her nasal cavity also demonstrated thick purulent secretions in the middle meatus bilaterally extending to the nasopharynx. Biopsies of the trachea, subglottis, and nasal septum demonstrated purulent, atypical squamous epithelium and necrotic tissue with bacterial colonies. Cultures from these sites were positive for methicillin-resistant *Staphylococcus aureus* (MRSA) and *Pseudomonas aeruginosa* but showed no fungal growth. As such, the patient was treated with intravenous vancomycin and cefepime in addition to steroids and nebulized treatments. Her breathing significantly improved during the course of her hospitalization and she was subsequently discharged with plans to continue outpatient intravenous antibiotics for an additional 2 months.

**Fig. 1:**
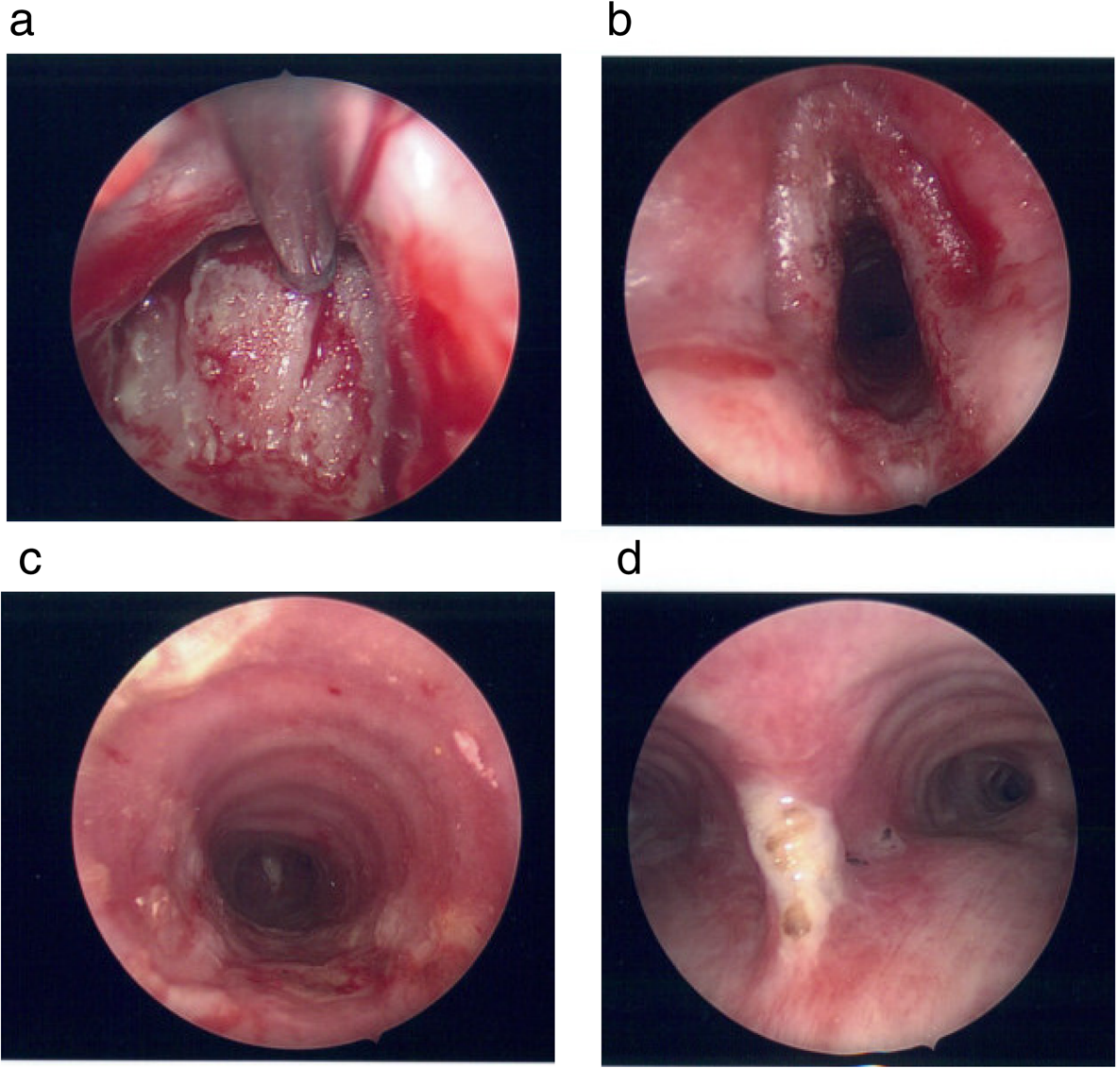
Endoscopic examination revealing widespread erythema with pseudomembrane formation in the (**a**) nasopharynx with (**b**) hyperemic, edematous vocal cords. (**c**) Patchy white pseudomembranes with accompanying erythema are seen in the trachea and track distally to the (**d**) bifurcation of the primary bronchi

**Fig. 2:**
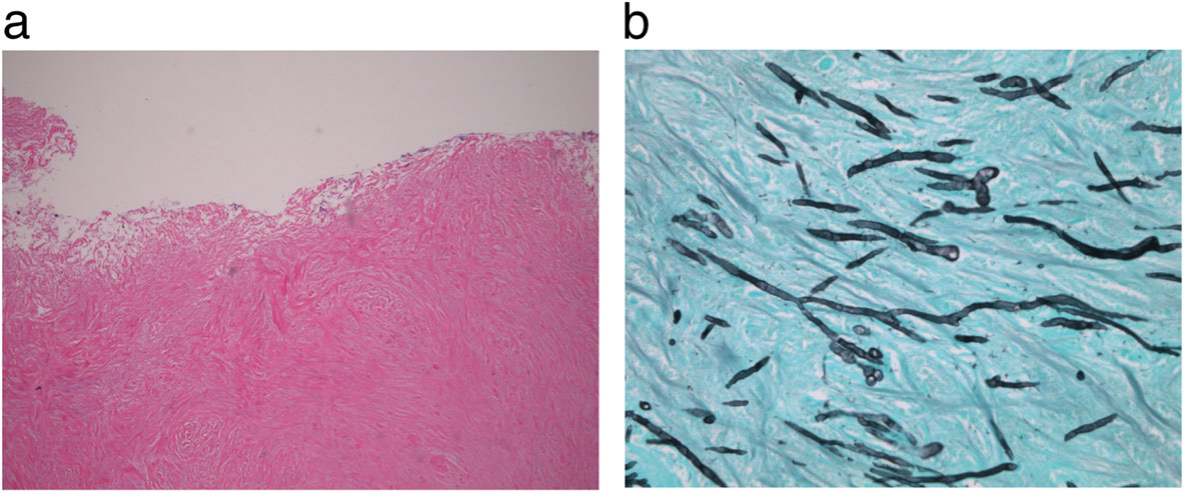
Tracheal biopsy demonstrating necrotic tissue on (**a**) HE staining. (**b**) GMS staining highlights branching fungal hyphae within the tissue specimen

Several months later, the patient returned for follow-up and was found to have similar upper airway symptoms despite completing her antibiotic regimen. She again underwent endoscopic examination that revealed multiple regions of white, friable pseudomembranous patches spanning her mid-trachea, subglottis, and nasopharynx (Fig. 1). Biopsies of these sites demonstrated diffuse tissue necrosis in the presence of extensive fungal hyphal infiltration (Fig. 2). Cultures from the trachea and sinuses grew *Aspergillus fumigatus*. Given a new diagnosis of invasive pseudomembranous aspergillosis, the patient was started on oral voriconazole (100 mg, twice daily) with close outpatient follow-up.

Over the following year, despite continuous antifungal treatment, the patient continued to experience tenacious, mucoid secretions, worsening dysphonia, nighttime pharyngitis, and frequent low-grade fevers. She required two separate inpatient admissions due to symptom escalation requiring endoscopic debridement of her trachea, pharynx, and nasal passages. Repeat tissue biopsies during this time period continued to demonstrate necrotic exudates in the presence of fungal hyphae with cultures continuing to grow pan-sensitive *Aspergillus fumigatus*. A formal immunological workup during this time was non-revealing with normal immunoglobulin levels, CD4+ T cell counts, absolute neutrophil counts, and complement levels. HIV testing was negative. Given her poor response to antifungal monotherapy, she was initiated on topical amphotericin B (100 mcg/ml) and budesonide (10 mcg/ml) nasopharyngeal rinses twice daily for symptomatic flares in addition to an increased dose of oral voriconazole (200 mg, twice daily).

The patient continued on this regimen for 2 months and was found to have marked improvement on repeat endoscopic examination, at this point 18 months after her initial diagnosis of invasive aspergillosis. Her larynx and subglottis had near normal appearance; her nasal cavities were dry bilaterally; her nasopharynx demonstrated mild scarring but otherwise demonstrated significantly less mucopurulence than on previous exams. However, in spite of this transient improvement, the patient again began developing upper airway symptoms over the following year, correlating with increased purulence in the nasal cavities along with the reformation of pseudomembranous, brown-mucoid layering in the nasopharynx seen on endoscopic exam. Increases in her voriconazole dosing to as high as 300 mg, twice daily were attempted but did not result in significant improvement and were further complicated by vision changes attributed to supratherapeutic voriconazole trough drug levels. Additionally, while the frequency of mechanical debridement had previously averaged once every four to 6 months, the patient began requiring nearly monthly endoscopic suctioning of her nasal passage and pharynx for symptom control.

Approximately 6 years after her initial diagnosis of invasive aspergillosis the patient continued to experience chronic symptoms including dysphagia, persistent dysphonia, and purulent secretions. Erosive changes to her posterior nasopharynx resulted in velopharyngeal insufficiency. Of note, serial chest x-rays during the course of her treatment never demonstrated abnormal parenchymal findings suggesting long-standing upper airway disease without progression to lower airway involvement. In the setting of persistently positive fungal hyphae seen on upper airway tissue biopsy, she was continued on oral antifungal therapy in combination with oral rinses and periodic mechanical debridement for symptomatic relief.

## Discussion and conclusions

Invasive aspergillosis of the respiratory tract is a rare but serious airway disease typically seen in immunocompromised patients. However, it is becoming increasingly clear that these infections can also occur in patients lacking the classic risk factors normally associated with underlying immunodeficiency. In the preceding case report, we presented the clinical course of an immunocompetent patient with clinical, microbiological, and histopathologic evidence of invasive pseudomembranous upper airway and tracheal aspergillosis persisting for many years in spite of longstanding treatment efforts. In our reading of the literature, this appears to be one of the most chronic cases of respiratory aspergillosis reported in an immunocompetent patient.

Patients found to have invasive *aspergillus*-related respiratory infections typically should have a formal immunological evaluation to rule-out primary and secondary causes of immunodeficiency. While different screening studies can be performed, enumeration of T cells and neutrophil counts are particularly important given their essential role in clearing fungal infections [9]. Given that these studies were all normal in our patient in the setting of her otherwise benign medical and family history, clinical suspicion for an underlying immunodeficiency was low and, in accordance with current practice guidelines [10], more advanced immunological testing was not pursued. However, other providers encountering similar case presentations could potentially consider additional testing to assess for functional defects in cellular immunity such as T-cell proliferation and cytokine responses [10].

The mechanism by which *aspergillus*-related respiratory infections develop in immunocompetent patients is poorly understood and difficult to study given the infrequency with which such cases occur and are reported. Interestingly, many of the comorbidities previously reported in immunocompetent patients diagnosed with invasive upper airway and tracheobronchial disease were notably absent in the case of our patient. D’Anza et al. [6] identified six total patients over a fifteen-year period with upper airway invasive aspergillosis in their multi-institutional retrospective study, of whom, four patients, while not overtly immunocompromised, had systemic comorbidities such as type 2 diabetes mellitus, chronic kidney disease, or congestive heart failure. In a separate study, Li et al. [5] identified fourteen patients who were diagnosed with invasive aspergillosis involving the tracheobronchial airways over a nine-year period at their institution. Of these, ten “low-risk”, immunocompetent patients were found to have chronic hepatitis B, chronic obstructive pulmonary disease (COPD), or diabetes at the time of diagnosis, all of which were absent in the case of our patient.

Given the absence of these previously described comorbidities, the question as to what initially predisposed our patient to her longstanding infection still remains. It does seem noteworthy that prior to presenting to our care, the patient was treated for mixed MRSA/pseudomonal bacterial sinusitis for approximately 2 years before developing evidence of aspergillosis on biopsy and culture data. Several case reports seem to suggest that chronic invasive aspergillosis may develop as a secondary infection following initial respiratory infections in immunocompetent patients. While these cases seem to be associated more often with viral etiologies such as influenza or even dengue [11, 12], it is possible that her longstanding bacterial infection may have resulted in anatomic injury, which in conjunction with the resulting local inflammatory milieu may have predisposed her to secondary infection by chronic *aspergillus* invasion in the absence of any other overt systemic predisposing risk factors. This is also potentially hinted at by the anatomical distribution of fungal disease seen in this case which appears to have mirrored the involvement of pre-existing invasive bacterial infection both histologically and on culture from the patient’s trachea and nasopharynx during her initial course. We also note that concurrent infection of the upper airway and tracheobronchial tree appears to be a particularly rare anatomic distribution for invasive respiratory aspergillosis, with infection of these sites typically occurring in isolation of one another or in combination with lung parenchymal involvement when the disease becomes particularly widespread and aggressive [5, 13].

Aside from the challenges in identifying the source of our patient’s infection, understanding her poor response to combinatorial systemic antifungal therapy and mechanical debridement has proven equally challenging. In accordance with current treatment guidelines for invasive aspergillosis, oral voriconazole was started and continued for the duration of her disease course [14–16]. However, titration of her medication dosing was complicated by findings of sub-therapeutic plasma drug levels on several occasions. Although poor medication adherence likely contributed to some of her low drug levels in the setting of such long-standing treatment, voriconazole dosing in general is known to be challenging due to large variability in its bioavailability resulting from drug-drug interactions, nonlinear saturable pharmacokinetics, liver disease, and genetic polymorphisms of CYP2C19 [16]. Attempts to subsequently increase her dosing caused episodic visual disturbances, a side effect associated with supratherapeutic drug levels [16], and resulted in short periods of time when the medication had to be discontinued to allow for symptom resolution. While the challenges in maintaining a consistent dosing regimen likely contributed to her disease course, the duration and refractory nature of her symptoms is still unexpected in a patient without underlying immunodeficiency, particularly in the setting of additional treatment with mechanical debridement.

When used in combination, voriconazole and endoscopic debridement of infected tissue typically results in excellent outcomes in immunocompetent patients. Looking specifically at cases of upper airway aspergillosis, the patients described by D’Anza et al. [6] all required just a single endoscopic procedure followed by treatment with voriconazole for an average of 4 months in order to have complete clinical and radiographic resolution of disease without recurrence. Wu et al. [2] described a similar strategy in the treatment of isolated tracheobronchial aspergillosis, with patients receiving oral or intravenous antifungal agents for an average of 25 days with intermittent bronchoscopic intervention. Even in this population, which had patients with serious underlying comorbidities including malignancy and tracheobronchial tuberculosis, resolution of disease was achieved in over 70% of patients. Interestingly, Li et al. [5] reported a much higher mortality rate in immunocompetent patients presenting with isolated tracheobronchial aspergillosis when treatment included only intravenous antifungal therapy without additional endoscopic debridement. However, these patients were, in general, a sicker cohort at the time of diagnosis and treatment, with many presenting with respiratory failure in the setting worsening disease progression involving the lung parenchyma.

Based on this previous work, the expectation at the outset of our patient’s treatment was that this combinatorial approach would be efficacious, particularly given her benign past medical history. However, it is notable that after years of active invasive infection, the patient had no radiographic involvement of the lung parenchyma on serial imaging, suggesting that while unable to completely resolve her disease, her treatment appears to have at least slowed her disease progression. The use of topical amphotericin B and steroid rinses, while more conventionally used for treatment of allergic fungal rhinosinusitis [17, 18] rather than for invasive disease specifically, was effective for symptom control in our patient and may be considered as an ancillary treatment option in other similar cases as long as care is taken to ensure that steroids are not administered as a monotherapy during treatment.

Although invasive upper airway aspergillosis is exceedingly uncommon in immunocompetent patients, such patients with chronic sinusitis and respiratory symptoms should be evaluated for the possibility of secondary invasive fungal infections, particularly those who fail to improve with appropriate antibiotic therapy. Although combinatorial voriconazole and endoscopic debridement is typically an efficacious treatment approach, some patients may continue to experience refractory symptoms, in which case continued aggressive treatment may potentially slow disease progression even if complete disease resolution cannot be achieved. Ultimately, this case highlights the possibility of refractory invasive aspergillosis in an immunocompetent patient and demonstrates the importance of further investigation into the pathogenesis of invasive fungal disease in the absence of overt systemic immunosuppression.

## Data Availability

Not applicable.

## Abbreviations

COPD: Chronic obstructive pulmonary disease
CT: Computed tomography
ESR: Erythrocyte sedimentation rate
HIV: Human immunodeficiency virus
MRSA: Methicillin-resistant *Staphylococcus aureus*
WBC: White blood cell
